# Heritability and Evolutionary Potential in Thermal Tolerance Traits in the Invasive Mediterranean Cryptic Species of *Bemisia tabaci* (Hemiptera: Aleyrodidae)

**DOI:** 10.1371/journal.pone.0103279

**Published:** 2014-07-23

**Authors:** Fang-Zhou Ma, Zhi-Chuang Lü, Ren Wang, Fang-Hao Wan

**Affiliations:** 1 State Key Laboratory for Biology of Plant Diseases and Insect Pests, Institute of Plant Protection, Chinese Academy of Agricultural Sciences, Beijing, P. R. China; 2 Center for Management of Invasive Alien Species, Ministry of Agriculture, Beijing, P. R. China; Zhejiang University, China

## Abstract

With advancing global climate change, the analysis of thermal tolerance and evolutionary potential is important in explaining the ecological adaptation and changes in the distribution of invasive species. To reveal the variation of heat resistance and evolutionary potential in the invasive Mediterranean cryptic species of *Bemisia tabaci*, we selected two Chinese populations—one from Harbin, N China, and one from Turpan, S China—that experience substantial heat and cold stress and conducted knockdown tests under static high- and low-temperature conditions. ANOVAs indicated significant effects of populations and sex on heat knockdown time and chill coma recovery time. The narrow-sense heritability (*h* ^2^) estimates of heat tolerance based on a parental half-sibling breeding design ranged from 0.47±0.03 to 0.51±0.06, and the estimates of cold tolerance varied from 0.33±0.07 to 0.36±0.06. Additive genetic variances were significantly different from zero for both heat and cold tolerance. These results suggest that invasive *B. tabaci* Mediterranean cryptic species possesses a strong ability to respond to thermal selection and develops rapid resistance to climate change.

## Introduction

The invasion of exotic species has accelerated along with the intensification of global climate change, the invasion of exotic species has accelerated. With respect to ecology, as one of the effects of global climate change, bioinvasion has deeply changed the structure and function of global ecosystems [1], [2]. With respect to evolution, biological invasion and global climate change have influenced selection pressures to various degrees and have even affected the evolutionary trajectories of organisms [3], [4].

The ability of an organism to remain active under extreme conditions is a critical component of fitness [5]. Thermal tolerance is an important factor in the distribution and range dynamics of insects [6]–[9]. Thermal tolerance has received much attention because it provides insight into how climate shapes variations in the ecology, distribution, hereditary and evolution of species [10]–[15]. The thermal tolerance of an alien species is crucial to its successful invasion [16], especially under conditions of climate warming [17]. Invasive species with high thermal tolerance could adapt faster under climate warming [18], [19], [20]. Determining thermal tolerance is an important first step in understanding the ways in which environmental variation affects fitness and, through changes in fitness, the dynamics of a given population. The differences in thermal tolerance among populations is an inheritable characteristic, representing a means by which insect evolution reacts to environmental stress [21], [22]. After an exotic species invades a new environment, new selection pressures may result in genetic variation within the species and may even result in the rapid adaptive evolution of tolerance traits [23], [24], [25]. Short-term evolutionary potential depends on the additive genetic variance in the population. The additive variance is often measured as heritability, the fraction of the total phenotypic variance that is additive. Thus, heritability is a common measure of evolutionary potential [26]. Therefore, understanding the variations in heritability and evolvability in thermal tolerance can help predict a species' future responses to climate change [5], [27], [28].

The whitefly *Bemisia tabaci* (Gennadius) is a complex species, containing at least 30 morphologically indistinguishable cryptic species, and undergoes rapid evolution [29]. The concomitant eruption of a group of plant viruses has caused considerable agricultural losses in many countries. The *B. tabaci* Mediterranean cryptic species (MED) can cause rapid and widespread invasions and is considered particularly dangerous [30], [31], [32]. First discovered in Yunnan Province, China, in 2003, *B. tabaci* MED has become the dominant pest species, expanding its range from tropical areas to frigid areas of China within ten years [36] and threatening to replace *Bemisia tabaci* (Gennadius) Middle East-Asia Minor1 (MEAM1) and indigenous species to become the new “super bug” [32]. The high heat-resistance ability of *B. tabaci* MED is one of the potential mechanisms underlying its invasive success [33], [34]. Thus, we considered MED to be an excellent research subject for rapid adaptive evolution.

The present study reports the trait means and variations in heat and cold resistance of *B. tabaci* MED to evaluate the evolutionary potential of these fitness components. By estimating the heritability of thermal tolerance, we show that *B. tabaci* MED exhibits high variance in its response to heat stress and has displayed modest evolvability after its successful invasion in China. As a first survey of the quantitative genetics of thermal resistance traits in the whitefly, we studied two introduced populations of the invasive *B. tabaci* MED whitefly distributed across China. We hypothesized that the broad distribution and ecological success of this invasive species could be explained by the high additive genetic variation in the physiological traits related to functional capacities.

## Materials and Methods

### Ethics Statement

*B. tabaci* is not a protected species in China; thus, no specific permissions were required for these locations and activities (e.g., the authority responsible for a national park or other protected area of land, the relevant regulatory body concerned with protection of wildlife, *etc*.).

### Whitefly Collection and Rearing

**Figure 1:**
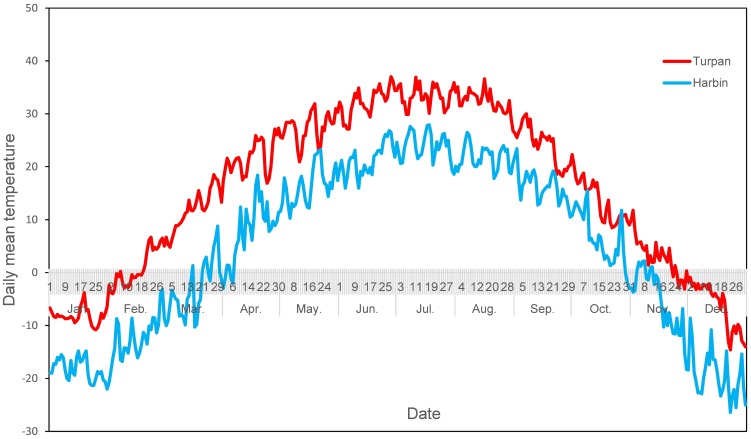
The daily mean air temperatures in Harbin and Turpan, China, in 2012. Data were obtained from the official website of the China Meteorological Data Sharing Service System (www.cdc.cma.gov.cn). The weather stations providing these data are located in Harbin, Heilongjiang (45.56°N, 126.70°E), and Turpan, Xinjiang (42.93°N, 89.13°E).

Whitefly eggs, nymphs and pseudopupae were collected from cotton, tomato and eggplant crops in fields near Harbin (45.56°N, 126.70°E), Heilongjiang Province, northern China, and Turpan (42.93°N, 89.13°E), Xinjiang Uighur Autonomous Region, western China. Harbin has the coldest weather and longest winters among the major Chinese cities. Due to the Siberian high-pressure system and its location above 45°N, the 24 h average temperature in January is only −18.4°C, and the annual mean temperature is 4.25°C, with the lowest temperatures dropping down to −42.6°C. *B. tabaci* can be found from July to November in the open field and overwinters in greenhouses in the winter months in Harbin [35]. As shown in Figure 1, *B. tabaci* MED experiences a daily mean air temperature (DMAT) range from 18°C to 26°C in July, and the DMAT decreases slowly in August and September in Harbin. Field populations of *B. tabaci* MED suddenly collapse when the temperature drops rapidly in mid- or late October, and field populations then move into greenhouses in Harbin in December; however, adult *Bemisia* can also be found in the open field during this time [39].

Turpan, one of China's four “oven cities”, is located at the other climatic extreme where summertime temperatures can soar to 49.6°C. Turpan has a unique temperate continental arid desert climate, with bright sunshine, high temperatures, and large day-night differences in temperature. The average maximum temperature is above 38°C from June to August, with more than 100 days experiencing a daily maximum above 35°C and 35–40 days with a daily maximum temperature above 40°C. *B. tabaci* MED can be found in cotton fields from April to December in Turpan. As shown in Figure 1, *B. tabaci* MED also experiences DMATs ranging from 18°C to 26°C in April, and the DMAT increases gradually in May and June. The DMAT can exceed 30°C in July and August, and *B. tabaci* MED expands its population distribution in the field during this period in Turpan. Field populations in Turpan decrease gradually in September, October and December with the decreasing DMAT.

Figure 1 shows that the temperature has a similar fluctuation trend during the year, but the daily mean temperatures are approximately 10°C lower in Harbin than in Turpan.

Therefore, we expected that whiteflies collected at these locations would be suitable to test our hypotheses regarding adaptation through thermal tolerance in an invasive species.

The Harbin and Turpan field populations (G0) were reared separately. Fifty field-inseminated females were collected from each population and used to establish iso-female lines in 100 specially designed rearing cages in the laboratory; the whiteflies were maintained at 26°C under a 14:10 hour light:dark cycle at 55–60% humidity. The cylindrical rearing cage was 13.5 cm long, with an internal fly-proof plastic screen (11.0 cm in diameter) made of transparent polypropylene to aid in ventilation. Each cage contained a single cotton seedling planted in a pot, which was filled with water. Species/cryptic species identification was confirmed in the G1 generation using the RAPD-PCR method [36] to ensure that all lines were in fact *B. tabaci* MED. This technique has proven to be a highly reliable method for distinguishing MED from other complex species [36]. Only the MED iso-female lines were kept and allowed to expand for an additional generation in the laboratory to ensure large population sizes for each line prior to setting up a mass breeding population. In the second generation (G2) after collection, mass-bred populations were founded with 10 females and 10 males from each of the 50 iso-female lines. The Harbin MED mass-bred population and the Turpan MED mass-bred population were kept separately at 26°C under a 14:10 hour light:dark cycle at 55–60% humidity in two 50×50×50 cm screened cages containing 16 cotton plants, and these populations were reared for three generations to ensure a large, effective population size.

### Experimental Design and Data Collection

To compare the heat and cold resistance between the populations and sexes, after three generations of mass rearing, four hundred individuals were randomly selected from each mass-bred population (G5) and scored for knockdown resistance (100 females and 100 males per heat stress and cold stress experiment) on the third day after emergence.

At the same time, we used a standard paternal half-sibling breeding design to estimate the genetic parameters for heat and cold tolerance of both of the Harbin and Turpan MED populations. For both populations (G5), one hundred virgins of parental males (sires) were randomly collected from each mass-bred population immediately after emergence using an air suction insect collector (described below). Each sire was placed in a rearing cage containing a single cotton seedling (with two cotyledons) and five virgin females (dams); the flies were allowed to mate and oviposit for 3 days before the adults were tested individually for knockdown resistance. When their offspring (G6) emerged, 4 females of each dam were collected, reared on cotton plants, mated with males and scored for knockdown resistance (two females for heat stress and 2 females for cold stress) when they were 3 days old. The breeding experiment was performed in the laboratory at 26°C under a 14:10 hour light:dark cycle at 55–60% humidity.

### Heat and Cold Stress

To test heat knockdown resistance, female individuals were carefully collected using an air suction insect collector (Mouth Aspirator with HEPA Filter, Model 612, John W. Hock Company, Gainesville, Florida, U.S.A., http://johnwhock.com/products/aspirators/mouth-aspirators).

To expose the insects to heat stress, one adult was confined in a 5 mL centrifuge tube containing a cotton pad through which it could breathe freely. The tubes were moved to a water bath controlled by a high-precision thermoregulator (CC-106A, Germany, Huber Kältemaschinenbau GmbH), which was set at a constant temperature of 45°C. We measured the interval between when the tube was placed in the water bath and when the whitefly lost control of its body and fell to the bottom of the tube.

To test cold resistance, we measured the time to recovery following a chill coma induced by cold shock. One adult, confined as described above, was exposed to −5°C through submersion into a 50% glycol solution cooled by a refrigeration bath circulator (K6-cc-NR, Germany, Huber Kältemaschinenbau GmbH) for a period of 10 minutes, and recovery time was measured at 26°C. Whiteflies assessed for heat and cold stress were tested on the same day.

### Statistical Analysis

Our data were generated from a standard paternal half-sibling breeding design (Lynch and Walsh, 1998). The animal model used to analyze the data was
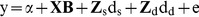
(1)where **X** is the design matrix for the fixed effect of run **B** and **Z**_s_ and **Z**_d_ are the design matrices for the random effects of sire and dam, respectively. The total phenotypic variance (σ^2^_P_) for the breeding design for the purpose of estimating genetic parameters was represented by
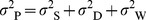
(2)where σ^2^_S_, σ^2^_D_, and σ^2^_W_, are the sire, dam and within-group level variance components, respectively. Variance and covariance components were estimated using the restricted maximum likelihood technique via the MIXED procedure in SAS (SAS Institute, Cary, NC). As we used a half-sib full-sib breeding design, the sire variance, σ^2^_S_, is one-fourth of the additive genetic variance (*V*_A_) [37], [38]. Thus, to estimate *V*_A_, we multiplied the sire variance by four.

The additive genetic variance for each trait was first estimated using a univariate model. Log likelihood ratio tests were performed, where the final model for each trait was compared to a model in which σ^2^_S_ was set to zero to determine whether the levels of additive genetic variance for each trait were significantly different from zero [38], [39], [40]. The phenotypic variance (*V*_P_) in knockdown resistance was computed using all known relationships among individuals. Then, we estimated the narrow-sense heritability for both traits. Narrow-sense heritability for each trait was estimated as the additive genetic variance (*V*_A_) divided by the total phenotypic variance (*V*_P_) [37], [41]. We conducted Student's t-tests to determine whether the variance components and heritability estimates differed significantly. Estimates of evolvability, *I*_A_, and the additive genetic coefficient of variance,
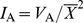
(3)(where 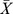 is the trait mean) [26], [42], were also computed for both traits.

For the population and sexual comparisons, ANOVAs were performed to evaluate the differences in the knockdown and recovery times, followed by the Tukey-Kramer multiple comparison test. The population (Harbin and Turpan) and sex (female and male) were used as the two factors. *P*<0.05 was considered to be statistically significant.

## Results

### Differences in Heat and Cold Resistance between the Populations and Sexes

Means, standard errors, and coefficients of variation (%) for heat knockdown time and chill coma recovery time of the Harbin and Turpan populations are listed in Table 1. The mean heat knockdown time was higher in the Turpan population than the higher latitude Harbin population; however, the mean chill coma recovery time was shorter in the Harbin population than the Turpan population (in both females and males). The mean heat knockdown times for the females of both the Harbin and Turpan populations were longer than those of the males, whereas the mean chill coma recovery times were shorter in males than in females. The variation of each trait was similar for both females and males, whereas the coefficients of variation varied considerably between the two traits. The rank order of variation was high for chill coma recovery time (ranging from 86.50% to 88.66%) and moderate for heat knockdown time (ranging from 39.14% to 41.05%).

**Table 1:** Mean, standard error (1 S.E.), and coefficients of variation for the thermal resistance of the Harbin and Turpan populations of *B. tabaci* MED cryptic species.

	Heat knockdown time, min			Chill coma recovery time, min	
		Mean ± S.E.	CV (%)	Mean ± S.E.	CV (%)
Harbin	Female	9.50±0.33	37.52	7.85±0.28	87.93
	Male	8.07±0.44	39.14	7.58±0.31	88.66
Turpan	Female	12.12±0.53	40.36	9.45±0.42	86.50
	Male	9.83±0.57	41.05	7.90±0.40	88.60

**Figure 2:**
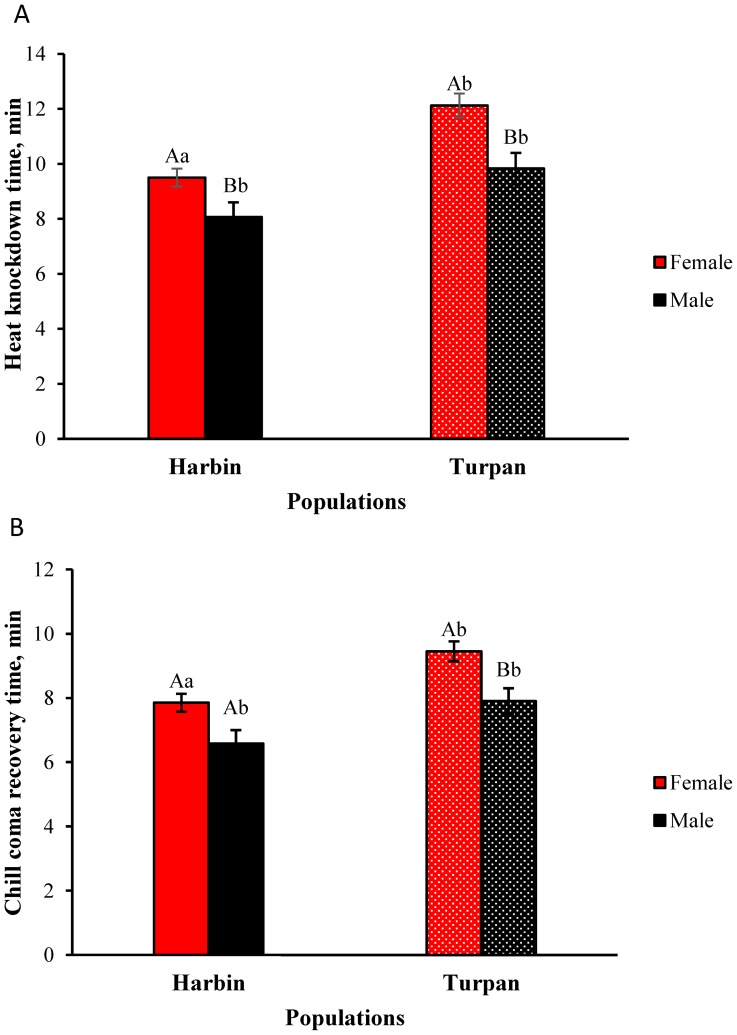
Heat knockdown time (A) and chill coma recovery time (B) (min) for the Harbin and Turpan populations of the *Bemisia tabaci* MED cryptic species. The numbers represent the means ±1 S.E.; N = 100 in all cases. Bars topped with a different capital letter indicate a significant difference between the females and males within the same population; the different lowercase letters indicate a significant difference in the same sex between the two populations of *B. tabaci* MED cryptic species (two-way ANOVA followed by *post hoc* Tukey-Kramer multiple comparison test, *P*<0.05).

Figure 2 shows the mean (±1 S.E.) heat knockdown and chill coma recovery times for the two populations of MED. At least 100 individuals of each sex from both populations were examined. An ANOVA indicated the significant effects of population (Harbin and Turpan) (*F*_1, 466_ = 20.50, *P*<0.001) and sex (*F*_1, 466_ = 18.97, *P*<0.001) on heat knockdown time (Figure 2a, Table 2), along with the significant effects of population (Harbin and Turpan) (*F*_1, 476_ = 21.77, *P*<0.001) and sex (*F*_1, 476_ = 17.92, *P*<0.001) on chill coma recovery time (Figure 2b, Table 2). There was no significant interaction between population and sex (*F*_3, 476_ = 14.56, *P* = 0.198) on heat knockdown time or chill coma recovery time (*F*_3, 476_ = 8.81, *P* = 0.418). Females showed a significantly higher heat resistance than males (*F*_1, 233_ = 8.126, *P*<0.001, for Harbin; *F*_1, 243_ = 8.198, *P*<0.001, for Turpan) and a significantly lower cold resistance (*F*_1, 233_ = 8.785, *P*<0.001, for Harbin; *F*_1, 243_ = 8.779, *P*<0.001, for Turpan).

**Table 2:** ANOVA table of heat knockdown time and chill coma recovery time of *B. tabaci* MED cryptic species.

Source	*df*	Mean Square	*F*	*P*
Heat knockdown time				
Populations	1	417.56	20.50	<0.001
sex	1	308.46	18.97	<0.001
Populations × sex	3	59.91	14.56	0.198
Total	466			
Chill coma recovery time				
Populations	1	36.21	21.77	<0.001
sex	1	27.34	17.92	<0.001
Populations × sex	3	5.90	8.81	0.418
Total	476			

### Heritability and Genetic Variance Components

The estimates of genetic variance were similar between the two protocols, as well as between the two populations (Table 3). Narrow-sense heritability estimates were significant for the heat knockdown times of both the Harbin and Turpan populations. The lower heritability for the heat knockdown time of the Turpan population seems to be driven by higher environmental variance, as significant levels of additive genetic variance were detected (Table 3). For chill coma recovery time, the narrow-sense heritability estimates were significant for both the Harbin and Turpan populations, and the lower heritability for the Turpan population seems to be driven by higher environmental variance (Table 3). This interpretation is supported by the mean standardized estimates of variation (Table 3); the coefficients of additive genetic variation and environmental variation were slightly larger and smaller for the Harbin population, respectively.

**Table 3:** Narrow-sense heritability (*h* ^2^), variance, and coefficient of variation components for heat knockdown time and chill coma recovery time (Harbin and Turpan populations) of *B. tabaci* MED cryptic species.

Mean/Variance*	Harbin population		Turpan population	
	Heat knockdown time, min	Chill coma recovery time, min	Heat knockdown time, min	Chill coma recovery time, min
*Mean* ± S.E.	9.73±0.38	7.79±0.44	11.99±0.38	9.68±0.44
*V*_A_ ± S.E.	9.80±0.002^1^	5.24±0.002^1^	11.69±0.002^1^	6.50±0.002^1^
*V*_E_ ± S.E.	13.58±3.13	11.23±2.67	18.69±4.22	14.07±2.85
*V*_P_ ± S.E.	19.21	14.56	24.87	19.69
*h* ^2^ ± S.E.	0.51±0.06*	0.36±0.06*	0.47±0.03*	0.33±0.07*
*I*_A_ × 100	10.35	8.63	8.13	6.94
*CV*_A_	0.322	0.294	0.285	0.263
*CV*_E_	0.143	0.185	0.130	0.150
*N*	946	957	929	936

Additive genetic variance (*V*_A_), environmental variance (*V*_E_), phenotypic variance (*V*_P_), narrow-sense heritability (*h* ^2^), evolvability (*I*_A_×100), coefficient of additive genetic variance (*CV*_A_) and coefficient of environmental variance (*CV*_E_) for heat knockdown time and chill coma recovery time. *N* =  sample size.

1 *P*<0.05 for log likelihood ratio test of significant differences from zero;

*P<0.05.

## Discussion

In our study, we compared the thermal tolerances of the Harbin and Turpan *B. tabaci* populations. The results indicated that genetic differences in thermal tolerance existed between the populations, with an increasing heat tolerance and a decreasing cold tolerance in the Turpan population compared with the Harbin population. Because Turpan experiences a very specific climate, characterized by a long-term, extremely high temperature, the Turpan population may be acclimated to the heat due to its exposure to a generally higher temperature compared with the Harbin population. By contrast, Harbin has the coldest temperatures and longest winter months compared with other major Chinese cities; thus, the Harbin population can survive a colder climate compared with the Turpan population. Our study demonstrated that field populations of *B. tabaci* MED cryptic species possessed a high heat tolerance in response to thermal stresses. This finding implies that there is a significant difference in the adaptive strategies to temperature between the Harbin and Turpan populations as a result of their different experiences with air temperature. Thus, we inferred that high, narrow-sense heritability for both heat and cold resistance is the direct driving force for the adaptive microevolution of *B. tabaci*.

Founder effects, genetic drift, and natural selection can all lead to genetic differentiation in populations of an invading species relative to the invader's source population [43]. In most cases, the alien populations have a high degree of spatial isolation, precluding gene flow among populations. In a laboratory selection experiment using velvetleaf (*Abutilon theophrasti*) and green amaranth (*Amaranthus retroflexus*), significant genetic shifts were observed in just four generations of selection under treatments involving different soil moisture levels, competitor diversity regimes, and intraspecific densities [44]. In the present study, thermal tolerance and phenotypic variance of the invasive whitefly *B. tabaci* were revealed using the static knockdown time method, which has been widely used in *Drosophila* experiments [45], [46]. Here, whitefly females of both study populations were more heat tolerant but less cold tolerant than males. These results partially agree with previous studies on MEAM1; however, a different experimental protocol was utilized in this study [47], [48], [49].

Differences in heat shock proteins (HSPs), especially *Hsp*70, which plays an important role in heat tolerance, might cause the observed differences between females and males. The optimum mRNA expression of HSP genes in females promotes a higher survival rate under heat shock conditions, and there are large between-species differences in HSP gene expression levels [50], [51], [52].

Given adequate genetic variability, invading species are often able to make rapid evolutionary adjustments. With regard to heat resistance, estimates of heritability in *D. melanogaster* based on knockdown time experiments at 39°C suggest values ranging from 0.03 to 0.11 [52] or up to 0.28 [42], whereas experiments at 38°C suggest values ranging from 0.14 to 0.22 [46]. These estimates might not be directly comparable to those obtained here, which ranged from 0.47 to 0.51 at 45°C, due to the difference in the temperature used to assess the trait as well as the different species examined. Nevertheless, the values obtained here showed that a substantial proportion of the total phenotypic variation in heat resistance was caused by additive genetic variation. Few studies have examined the genetic variance of cold resistance [53]–[56]. Estimates of heritability on recovery from a chill coma at 0°C suggest values ranging from 0.01 to 0.38 [55]. Our estimates of heritability for *B. tabaci* MED based on chill coma recovery time at −5°C (0.33 to 0.36) also indicated that a substantial proportion of the total phenotypic variation was caused by additive genetic variation.

The values of the evolvability measure, CV_A_, which reflects the potential shift in a trait relative to the trait mean, were 0.285–0.322 and 0.263–0.294 for heat knockdown time and chill coma recovery time, respectively. Consequently, the evolvability of cold resistance indicated a stronger evolutionary response than that of heat resistance. For heat resistance, due to the influence of the experimental protocol on estimates, previous estimates of evolvability for *D. melanogaster* based on knockdown time at 38°C suggest broad values, ranging from 0.02 to 14.53. Estimates of additive genetic variance and measures of evolvability were higher when *D. melanogaster* flies were exposed to a static stress rather than a ramping (when temperature was increased to an upper limit) stress when genetic variances were standardized according to the trait means [46].

Several studies have investigated the heritability and evolutionary potential of thermal tolerance traits in invasive species. In the current paper, we provide data for the invasive Mediterranean cryptic whitefly species (*B. tabaci*), which is now widely distributed across China. By measuring the narrow-sense heritability (*h* ^2^) of thermal tolerance traits and because of the high CV_A_ value of knockdown time, we show that natural *B. tabaci* MED cryptic species exhibit a high adaptive potential against both heat and cold stress. The *h* ^2^ and CV_A_ values of heat tolerance traits were higher than the cold tolerance traits in natural MED, which indicated the whiteflies were more able to respond to heat selection in field MED populations than cold selection. We suggest that MED expansion is likely to be favored by climate warming and that the high evolutionary potential of heat tolerance has enabled its invasion into mid- and high-latitude areas.
